# Sarcoid aortitis presenting as an ascending aortic mass mimicking intramural haematoma and complicated by retrograde aortic dissection: a case report

**DOI:** 10.1093/ehjcr/ytag180

**Published:** 2026-04-13

**Authors:** Mohamed Salah Shehata, Abdalla Elagha

**Affiliations:** Cardiovascular Department (Kasr-Alainy Hospital), Cairo University, 1 Gamaa Street, Giza 12613, Cairo, Egypt; Cardiovascular Department (Kasr-Alainy Hospital), Cairo University, 1 Gamaa Street, Giza 12613, Cairo, Egypt

**Keywords:** Case report, Sarcoidosis, Aortitis, Aortic dissection, Cardiac MRI, Intramural haematoma, Granulomatous inflammation, Cardiac sarcoidosis

## Abstract

**Background:**

Sarcoidosis is a multi-system disorder characterized by non-caseating granulomatous infiltration, commonly affecting the lungs. Cardiac involvement is rare but potentially life-threatening. Aortitis in sarcoidosis is extremely rare and scarcely reported in the literature.

**Case summary:**

We report a unique case of a 51-year-old female patient with multi-system extra-cardiac sarcoidosis who was referred for cardiac magnetic resonance imaging (CMR) to exclude cardiac sarcoid involvement. Imaging revealed a retrograde Stanford Type A aortic dissection with an infiltrative mass engulfing the ascending aorta, initially presumed to represent an intramural haematoma (IMH). However, detailed CMR tissue characterization demonstrated findings inconsistent with IMH and more indicative of inflammatory activity of the ascending aortic wall.

**Discussion:**

This case highlights the rare presentation of sarcoid aortitis masquerading as IMH on initial imaging, underscoring the importance of advanced tissue characterization in atypical aortic pathology and the potentially catastrophic complications of granulomatous vascular involvement.

Learning points

- Sarcoid aortitis represents an exceptionally uncommon manifestation of sarcoidosis.
- Granulomatous inflammation of the ascending aorta can mimic intramural haematoma on imaging.

## Introduction

Sarcoidosis is a multi-system, non-caseating granulomatous disorder. Multiple factors contribute to the pathophysiology of sarcoidosis, including infection, genetic predisposition, and environmental factors, predominantly involving the lungs, lymphatic system, liver, and skin.^1^ Cardiac sarcoidosis (CS) occurs in ∼5% of sarcoidosis cases but may be underdiagnosed.^2^ Approximately 25% have asymptomatic, clinically silent cardiac involvement verified by autopsy or imaging studies.^3^ Symptomatic CS can manifest by conduction abnormalities, ventricular arrhythmias, heart failure, or sudden cardiac death. Aortic involvement in sarcoidosis, especially as aortitis, is extremely rare.^4^ Granulomatous inflammation of the aortic wall may mimic other aortic pathologies, including intramural haematoma (IMH), vasculitis disorders, or neoplastic infiltration. The presence of sarcoid aortitis predisposing to or complicating aortic dissection has not been well documented.

## Summary figure

**Figure:**
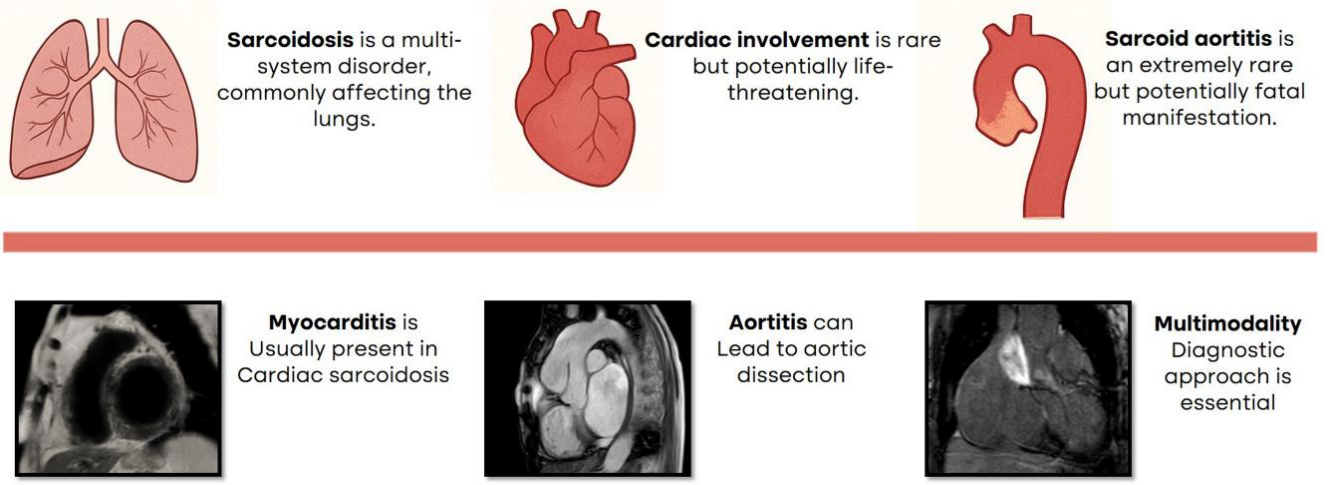


## Case presentation

**Figure 1:**
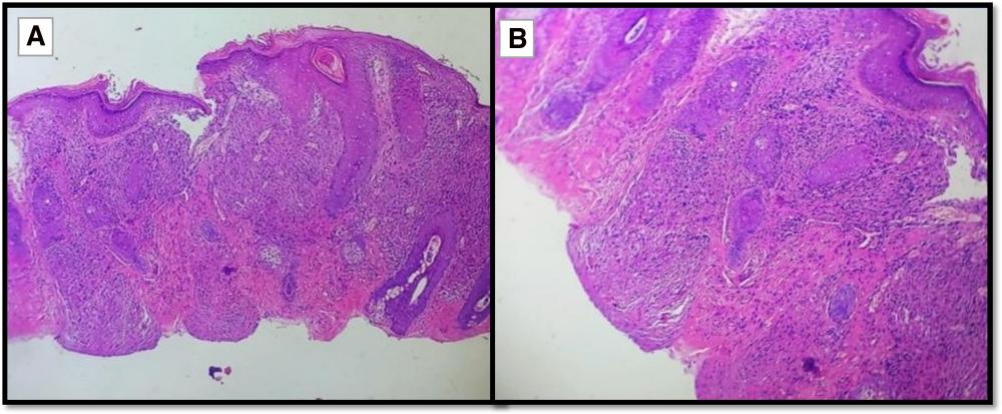
Shave skin biopsy from a papule showing collection of epithelioid histiocytes with minimal lymphocytic infiltrate (*A*). Multiple discrete predominantly non-caseating granulomas (*B*). Stratum corneum showed impact corneum with parakeratosis with no neutrophils or fungal hyphae seen and unremarkable epidermis findings (*A* and *B*).

A 51-year-old female recently diagnosed of systemic sarcoidosis with pulmonary, hepatic, and cutaneous involvement presented for evaluation of potential cardiac involvement. The patient had no known history of hypertension, connective tissue disease, or prior aortic pathology. Her condition had been diagnosed 3 months prior via histologic confirmation of *non-caseating granulomas from a nasal papule biopsy* (*Figure 1*). She had previously been managed with systemic corticosteroids with partial disease control.

At presentation, the patient was complaining of shortness of breath, palpitations, and dizziness. Trans-thoracic echocardiography (TTE) revealed bi-atrial dilatation, significant atrioventricular (AV)-valvular regurgitation, and small ventricular cavities with restrictive physiology. Cardiac biomarkers, including troponin and N-terminal pro B-type natriuretic peptide, were above normal limits.

Given the systemic nature of sarcoidosis, a *cardiac magnetic resonance imaging (CMR)* was ordered to evaluate for cardiac involvement. Cardiac magnetic resonance imaging demonstrated multiple abnormal findings not just limited to myocardial integrity and structure but also aortic abnormalities.

**Figure 2:**
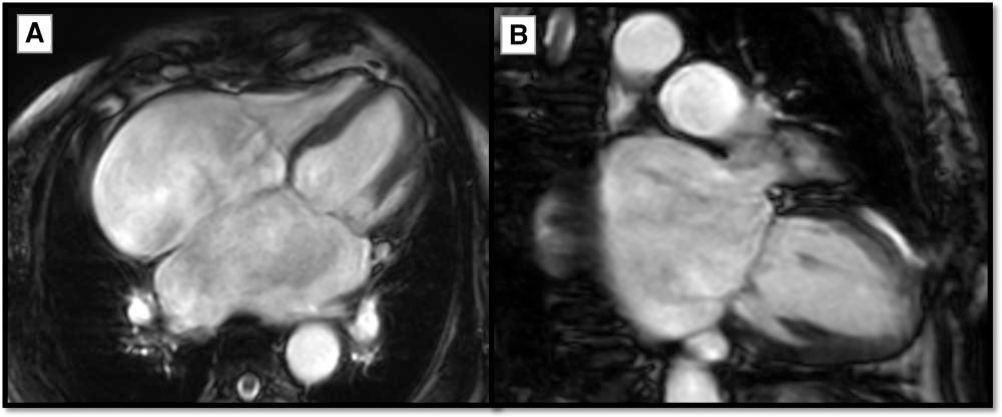
Cardiovascular magnetic resonance imaging steady state free precession (SSFP) images. (*A*) Four-chamber view SSFP image showing marked bi-atrial dilatation, small ventricular cavities, and restrictive configuration of the heart. (*B*) Two-chamber view SSFP image showing hugely dilated left atrium and small left ventricular cavity.

**Figure 3:**
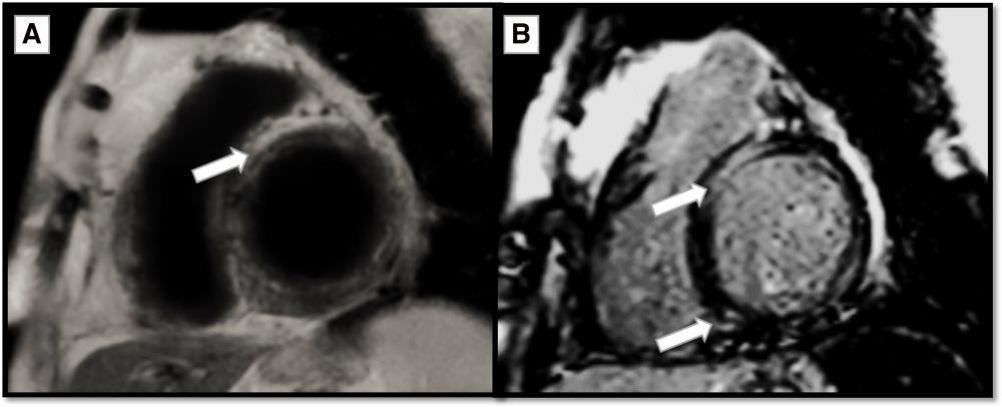
(*A*) Short-axis view T2-weighted image showing increased signal intensity of the basal anteroseptal segment; indicative of myocardial oedema (thick arrow). (*B*) Short-axis view late gadolinium enhancement image showing hyperenhancement of multiple myocardial segments; indicative of myocardial fibrosis (thick arrows). Both findings fulfil the original Lake Louise criteria of active myocarditis.

Myocardial assessment by *cine images confirmed the restrictive physiology of the heart, marked bi-atrial dilation, and severe AV-valvular regurgitation* (*Figure 2*). Moreover, tissue characterization by *T2-wieghted images revealed a high signal intensity (SI) of multiple segments* indicating the presence of active myocardial oedema. While, *late gadolinium enhancement (LGE) images* demonstrated multiple patchy areas of hyperenhancement in the mid-myocardial and subepicardial layers, involving the apical lateral, basal anteroseptal, and basal inferolateral segments, with additional hyperenhancement at the anterior and inferior right ventricular insertion points at the apical, mid-, and basal levels (*Figure 3*). Findings are fulfilling the *Lake Louise criteria of active myocarditis with a pattern suggestive of sarcoid-induced myocarditis*.

**Figure 4:**
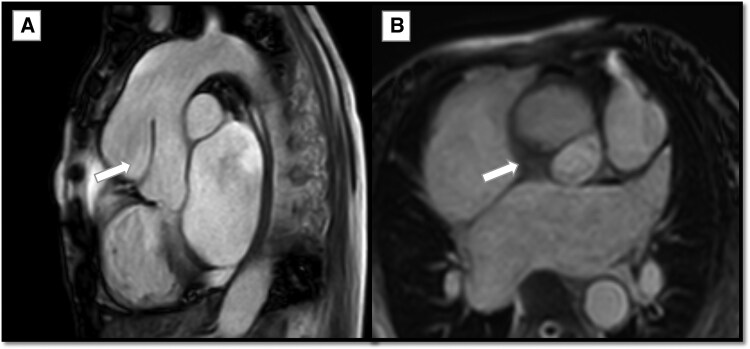
Cardiovascular magnetic resonance imaging SSFP. (*A*) Sagittal cut SSFP image showing retrograde Stanford Type A aortic dissection (thick arrow). (*B*) Axial cut SSFP image showing circumferential, crescentic soft tissue mass encasing the ascending aorta just above the level of sinotubular junction with true and false lumens.

Aortic assessment revealed a retrograde Stanford *Type A aortic dissection (RTAD)* with a circumferential, crescentic soft tissue mass encasing the ascending aorta. The mass measured ∼11 mm at maximal thickness in axial images, extending from the sinotubular junction to just below the proximal aortic arch. The lesion spared the aortic root with no affection of the coronary artery ostium. Initially presumed to represent an intramural hematoma due to its crescentic configuration and aortic wall involvement (*Figure 4*).

**Figure 5:**
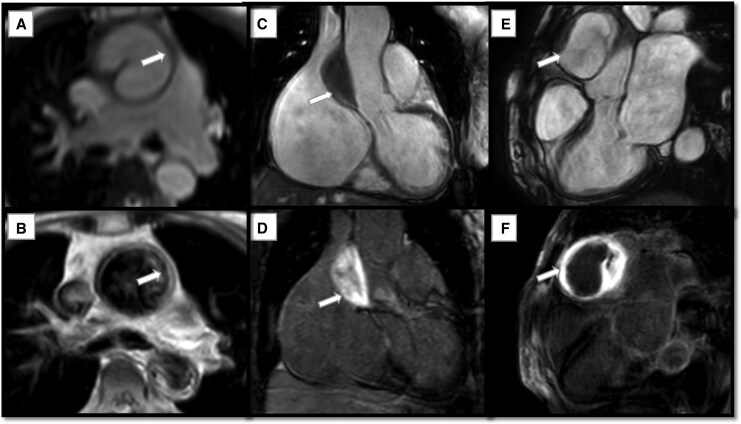
(*A* and *B*) Axial cut SSFP image showing thickened aortic wall (*A*). Axial cut T2-weighted image at the same level showing high signal intensity of the aortic wall (*B*). (*C* and *D*) Coronal cut SSFP image showing the mass encasing the ascending aorta extending from the sinotubular junction to just below the proximal aortic arch (*C*). Coronal cut late gadolinium enhancement image showing avid enhancement of the mass (*D*). (*E* and *F*) Three-chamber view SSFP image showing circumferential mass encasing the ascending aorta (*E*). Three-chamber view late gadolinium enhancement image showing avid enhancement of the mass with mid-myocardial basal anteroseptal hyperenhancement (*F*).

However, further tissue characterization sequences on CMR revealed the following:

- *High SI on T2-weighted sequences* of the aortic wall, suggestive of active inflammation and oedema
- *Avid LGE* of the aortic wall mass, inconsistent with the signal characteristics of IMH or aortic atheroma (*Figure 5*)

These imaging features supported by the clinical context were more consistent with the *inflammatory process of the aortic wall*, i.e. *sarcoid aortitis*, rather than an acute IMH.

The patient underwent emergent ascending aortic replacement with a Dacron graft. Intraoperative findings included a thickened and friable ascending aortic wall with perivascular adhesions. Histopathologic examination of the resected aorta demonstrated non-caseating granulomatous inflammation with multinucleated giant cells, consistent with sarcoid aortitis. No evidence of necrotizing vasculitis or infectious aetiology was seen. Microbiological cultures and polymerase chain reaction for tuberculosis and fungal organisms were negative.

Postoperative recovery was uneventful. The patient was restarted on high-dose corticosteroids and initiated on methotrexate as a steroid-sparing immunosuppressant and anti-failure regimen.

## Discussion

This case illustrates an extremely rare presentation of *sarcoid aortitis* mimicking an IMH on initial imaging, complicated by a *retrograde aortic dissection*. Aortitis in sarcoidosis is extremely uncommon, with only sporadic case reports in the literature. The pathogenesis is thought to be due to granulomatous inflammation infiltrating the aortic media, leading to weakening of the aortic wall, predisposing to dissection or aneurysmal formation.

An ascending aortic mass with crescentic morphology can have several differential diagnoses by CMR. *Intramural haematoma* typically presents as a crescent-shaped deformity within the aortic wall with low signal in T1, T2, and LGE images. *Vasculitis or inflammatory aortitis* often demonstrates a thickened aortic wall with oedema and enhancement, leading to high T2 signal. *Neoplastic infiltration* usually shows suppressed T1 and T2 signals with heterogeneous enhancement on LGE and may invade adjacent structures. In this case, *CMR tissue characterization*—including wall thickening, high T2 signal, and homogeneous LGE—was decisive in identifying the inflammatory nature of the lesion and differentiating it from haematoma or neoplastic processes. These findings, however, are based on a single case and should be interpreted cautiously in similar presentations.

Importantly, the granulomatous infiltration of the aortic wall in this case directly preceded and likely predisposed to the catastrophic vascular event. The structurally compromised aortic wall was vulnerable to dissection, despite the absence of conventional risk factors, such as hypertension or connective tissue disease.

Histological confirmation remains the gold standard for diagnosing sarcoid aortitis, but it is seldom achievable without surgical intervention. Consequently, a combination of non-invasive imaging, clinical context, and histological evidence of systemic sarcoidosis is essential for establishing the diagnosis.

Management of sarcoid aortitis typically involves *immunosuppressive therapy*, primarily corticosteroids, with adjunctive agents in refractory cases. In cases complicated by dissection or aneurysm, *surgical intervention is mandatory*.

## Conclusion

Sarcoid aortitis is an extremely rare but potentially fatal manifestation of systemic sarcoidosis. This case underscores the diagnostic challenges posed by atypical imaging presentations and the critical role of advanced CMR in guiding diagnosis. Prompt recognition of aortic involvement and aggressive management are essential, particularly given the risk of life-threatening complications, such as aortic dissection. A high index of suspicion should be maintained for vascular involvement in patients with systemic sarcoidosis presenting with unusual aortic findings.

## Data Availability

The datasets used and/or analysed during this study are available from the corresponding author upon reasonable request.

## Lead author biography

Dr Mohamed Salah Shehata is a cardiology specialist and senior cardiovascular magnetic resonance (CMR) fellow at Alkasr Al Ainy Medical School, Cairo University. His primary research interests include advanced CMR techniques, coronary microvascular circulation, and the CMR-based assessment of reperfusion strategies in acute coronary syndromes. He is actively engaged in both clinical practice and research, with a focus on improving diagnostic and prognostic applications of cardiac imaging.

## Notes

**Consent:** Written informed consent was obtained from the patient for the publication of this manuscript and any accompanying images or data. The authors confirm that all procedures involving human participants were conducted in accordance with the ethical standards of the responsible institutional and/or national research committee and with the principles outlined in the Committee on Publication Ethics (COPE) guidelines. All reasonable efforts have been made to protect patient anonymity and confidentiality.
